# Responsive Vagus Nerve Stimulation for Drug Resistant Epilepsy: A Review of New Features and Practical Guidance for Advanced Practice Providers

**DOI:** 10.3389/fneur.2020.610379

**Published:** 2021-01-15

**Authors:** Breanne Fisher, Julie A. DesMarteau, Elizabeth H. Koontz, Seth J. Wilks, Susan E. Melamed

**Affiliations:** ^1^Division of Neurology, Ann & Robert H. Lurie Children's Hospital of Chicago, Feinberg School of Medicine, Northwestern University, Chicago, IL, United States; ^2^Department of Neurosciences, Medical University of South Carolina, Charleston, SC, United States; ^3^Neuromodulation Division, LivaNova, Houston, TX, United States; ^4^Division of Neurology, Children's Hospital of Philadelphia, Philadelphia, PA, United States

**Keywords:** VNS Therapy®, neuromodulation, closed-loop stimulation, dosing, programming

## Abstract

Vagus nerve stimulation (VNS) is a safe and effective therapy that has been available for over 20 years for adults and children with drug resistant epilepsy (DRE). Since U.S. Food and Drug Administration approval in 1997, VNS has been implanted in over 100,000 patients including over 30,000 children as an adjunctive therapy in reducing the frequency of seizures in patients 4 years of age and older with focal seizures that are refractory to antiseizure medications. VNS Therapy® has evolved over time and currently offers closed-loop, responsive stimulation as well as advanced features that streamline dosing and patient management. Advanced Practice Providers (APPs) such as nurse practitioners, physician assistants and clinical nurse specialists are integral in a comprehensive healthcare team, and dedicated VNS clinics have formed at comprehensive epilepsy centers across the world that are often managed by APPs. This approach improves access, education, and continuity of care for those with VNS or those considering VNS. Here we provide a review for APPs on the VNS Therapy® system focused on new features, dosing, and troubleshooting strategies with the goal to provide guidance to those managing VNS patients.

## Introduction

The field of bioelectric neuromodulation is growing as a complementary intervention to pharmaceuticals. While pharmaceuticals deliver a dose of small molecules that are circulated throughout the body, neuromodulation devices deliver targeted doses of electrical stimulation to the body's neural circuitry which can be implemented to treat a wide array of disorders. New terms for this field such as bioelectric medicines and electroceuticals highlight the similarities between neuromodulatory and pharmaceutical interventions. Despite these similarities, neuromodulation offers key advantages in terms of reversible, targeted therapy with minimal long-term side effects and adherence issues.

Communication in the form of electrical signals travels bidirectionally between the peripheral and central nervous system with the vagus nerve being one of the largest and longest transmission lines connecting much of the body's internal organs to the brain. Therefore, it is no surprise that modulation of the vagus nerve is the most studied and targeted peripheral nerve in the field of neuromodulation. In the past decade, over 2,500 articles on vagus nerve stimulation (VNS) have been published, and VNS has been investigated in a wide range of disorders including epilepsy, depression, heart failure, stroke, tinnitus, inflammation, and more.

Vagus nerve stimulation therapy received U.S. Food and Drug Administration (FDA) approval as an adjunctive treatment for drug resistant epilepsy (DRE) in 1997 as well as treatment resistant depression in 2005. Over the past two decades, VNS Therapy® has been implanted in over 100,000 people with DRE, and a large breadth of evidence has since formed confirming the safety and efficacy of VNS in DRE. Neuromodulation therapies have been on the rise, and VNS Therapy® for DRE has evolved into a smarter and more versatile therapy. Patients and healthcare providers can now benefit from closed-loop, responsive VNS and advanced features that enable more personalized therapy and streamlined dosing.

Vagus nerve stimulation clinics have formed at epilepsy centers across the world which counsel, manage and treat DRE patients with VNS as well as DRE patients that may benefit from VNS. Advanced Practice Providers (APPs), which includes nurse practitioners, physician assistants and clinical nurse specialists, have become increasingly integral in the delivery of neurological care (1) including that in epilepsy (2). Advanced Practice Providers often play a key role in VNS clinics by improving access and promoting continuity of care through patient and family education, managing patient therapy and providing essential follow-up care to VNS patients. Kennedy and Schallert published a nursing review on VNS in 2001 summarizing VNS Therapy®, guiding nurses in the daily treatment of patients with VNS devices (3). This article provides an update on the Kennedy and Schallert nursing review by detailing the approach to a dedicated VNS clinic, a discussion of the closed-loop nature of VNS including improved understanding of dosing, advanced features, and troubleshooting techniques that aid in improved patient management by APPs practicing in epilepsy clinics. The goal is to provide guidance to APPs new to VNS Therapy® as well as those already managing VNS patients or clinics.

## Traditional VNS

The VNS Therapy® system includes a pulse generator surgically implanted below the left clavicle which connects to a wired lead that is tunneled to the neck and terminates with electrodes wrapped around the left cervical vagus nerve. The generator sends electrical pulses through the lead and electrode to the vagus nerve. The goal of electrical stimulation is to activate vagal afferent fibers that project to the nucleus tractus solitarii (NTS) which sends signals to other brainstem nuclei including the raphe nucleus (serotonergic neurons) and locus coeruleus (noradrenergic neurons). Neuromodulation of electrical and chemical signaling through these brain regions is thought to be responsible for the anti-seizure effect of VNS (4).

Traditional VNS includes two modes of stimulation: normal mode (open-loop) and magnet mode (on-demand). Normal mode stimulation is the primary operating mode in which the device continually cycles between on and off periods (e.g., 30 s on and 5 min off). Magnet mode stimulation allows the patient or caregiver to deliver on-demand stimulation triggered by swiping a magnet over the area of the implanted pulse generator.

Five clinical trials (E01–E05) evaluating traditional VNS were conducted between 1988 and 1997 which enrolled a total of 454 patients with DRE. The two randomized, blinded, active controlled trials, E03 and E05, compared a cohort receiving traditional VNS (high-stimulation group) to a cohort receiving presumably subtherapeutic VNS (low-stimulation group). After 3 months, the high-stimulation group had a significantly higher mean seizure frequency reduction than the low-stimulation group [24.5 vs. 6.1% in the E03 trial (5) and 27.9 vs. 15.2% in the E05 study (6)]. Long-term, open-label follow-up of the subjects enrolled in the E01–E05 clinical studies showed seizure reduction continued to improve over time with mean seizure frequency reductions of 44% after 2–3 years of VNS Therapy® (7).

More recently, a retrospective analysis of 436 patients treated with traditional VNS showed a mean seizure reduction of 55.8% after a mean follow-up of 5 years (8). Of those patients with > 10 years of follow-up (*n* = 65), seizure-reduction continued to improve with follow-up duration to 75.5% after 8 years (9). A systematic review of 2,869 patients across 78 studies and VNS registry data from 5,554 patients, revealed ~60% of patients achieved a ≥50% seizure reduction after 2–4 years, with a seizure-freedom rate of 8% (10).

In addition to improvements in seizure control, VNS has shown to improve quality of life. In the VNS Therapy Patient Outcome Registry, quality of life metrics were assessed by providers in over 5,000 patients at various follow-up visits. Providers reported improvements in alertness (58–63% of patients, range over follow-up period), post-ictal state (55–62%), cluster seizures (48–56%), mood change (43–49%), verbal communication (38–45%), school/professional achievements (29–39%), and memory (29–38%) (11). Reports of improvements in mood associated with VNS (12, 13) led to the investigation of VNS in treatment resistant depression (14) which was later FDA approved in 2005.

## Modern VNS

Acute benefits of on-demand VNS were observed by manually swiping a magnet over the pulse generator prior to or during a seizure (15). Therefore, closed-loop or responsive VNS was developed to automatically deliver VNS in the absence of the ability to perform a magnet swipe. Since ~82% of people with epilepsy experience increased heart rate during seizures, defined as ictal tachycardia (16), a cardiac based seizure detection algorithm was implemented. Although this detection system does not have the ability to identify ictal events, it uses heart rate increases as a surrogate marker for seizures. This third mode of VNS is known as AutoStim and is available with generator models 106 AspireSR® and 1000 SenTiva®.

AutoStim was first clinically evaluated with the model 106 AspireSR® generator in a multicenter E-36 study in Europe (17) and E-37 study in the United States (18). Combined, 51 patients were implanted with VNS and observed in the epilepsy monitoring unit with AutoStim enabled. A total of 155 seizures were recorded from 32 patients. AutoStim was triggered during 48 of the 155 (31%) seizures. During the period of AutoStim being delivered, 29/48 (60%) of seizures ended. Responder rates at 12 months were 30% (8/27) in the E-36 study and 50% (10/20) in the E-37 study.

The AutoStim feature works by comparing the heart rate over the last 10-s (foreground) with the heart rate over the previous 5-min (background). Triggering of AutoStim occurs when the foreground heart rate exceeds the background heart rate by a programmer-defined threshold which can be set between 20 and 70% in 10% increments. The AutoStim threshold setting impacts the sensitivity of triggering an automatic stimulation based on heart rate changes, the rate of nonseizure-related automatic stimulations, and latency in triggering stimulation relative to the seizure onset. The lowest AutoStim threshold of 20% is associated with the highest sensitivity, capturing ~80% of seizures, and highest false positive rate of ~7 nonseizure-related stimulations per hour. The AutoStim feature is designed to limit the patient from getting stimulation at an unsafe duty cycle. As AutoStim threshold increases, sensitivity decreases as well as the number of false positives. Data from the E-36 and E-37 studies showed that lower AutoStim thresholds corresponded to shorter latency between seizure onset and triggering of AutoStim, and shorter latencies were associated with shorter seizure durations. Therefore, lower AutoStim thresholds have the potential to detect more elevations in heart rate associated with seizures with an earlier response time but also deliver more nonseizure-related stimulations which will increase the overall duty cycle (19). In the E-37 study, AutoStim was associated with a 5% increase in duty cycle (11% without AutoStim compared to 16% with AutoStim) (18).

**Figure 1:**
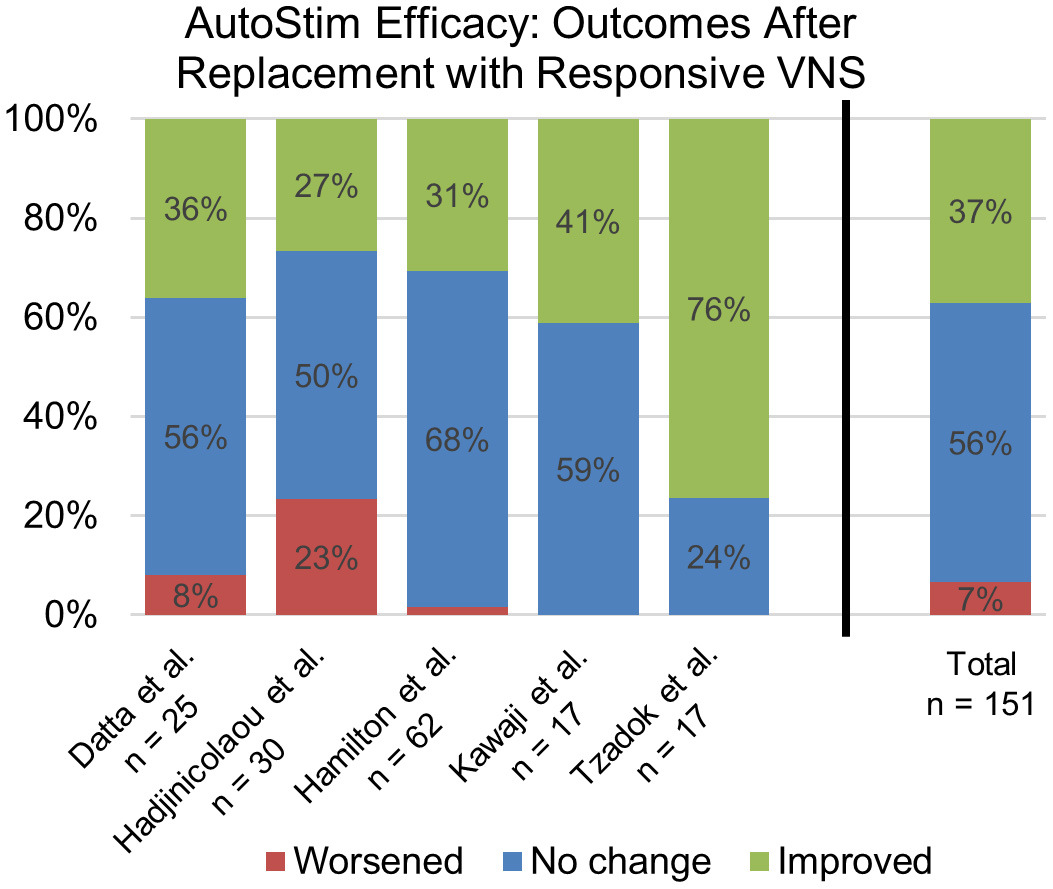
Summary of data reported on patients with traditional VNS after replacement with a modern VNS generator with AutoStim enabled (20–24). The bar graph on the far right represents collated data from all five studies.

Five separate studies (20–24) reported long-term outcomes in patients starting on AutoStim, either via a new VNS implant or a replacement with an AutoStim capable generator. Combining these datasets resulted in 80 patients who received new VNS implants and 151 patients receiving a generator replacement. Sixty percent of patients newly implanted with AutoStim VNS enabled were considered responders with a ≥50% reduction in seizure frequency after a mean follow-up period of 13 months. For those on traditional VNS receiving a generator replacement with AutoStim enabled, more than one-third of patients experienced additional improvement in seizure frequency by adding AutoStim (Figure 1).

## Dosing VNS Therapy®

Vagus nerve stimulation is an electroceutical, and dosing is similar to that of a pharmaceutical. Instead of dosing in milligrams, VNS is dosed in milliamps (mA) of electrical current, and it is often true that higher output currents increase the likelihood that vagus nerve fibers will be activated (25, 26). Similar to a medication, the output current of VNS needs to be titrated up to achieve a therapeutic effect. Titration typically begins 2 weeks after implantation giving the patient some time to heal from surgery. The goal of titration is to optimize output current to a therapeutic level that is well-tolerated by the patient. Suggested programming involves starting at a normal mode output current of 0.25 mA and increasing output current by 0.25 mA every 2 weeks to a maximal tolerated current, typically with a goal of 1.5–2.25 mA. This is often considered the “therapeutic dose.” The speed of titration can vary and depends on the comfort level of the healthcare provider, patient, and caregiver. Patient tolerability relates to the degree at which the patient feels sensations or experiences side effects associated with VNS. The most common stimulation associated side effects are hoarseness and voice alterations (7, 27). Paresthesia, cough, and shortness of breath are the next most common side effects. Other less common side effects include dyspepsia (indigestion), vomiting, increased incidence of obstructive sleep apnea, and hiccups. It is important that increases in output current are conducted at a rate that is tolerable and comfortable to the patient, however, patients are known to have better outcomes when they achieve a dose of 1.5–2.25 mA (26, 28). Over time, patients better tolerate VNS and the side effect profile diminishes (7, 27).

**Figure 2:**
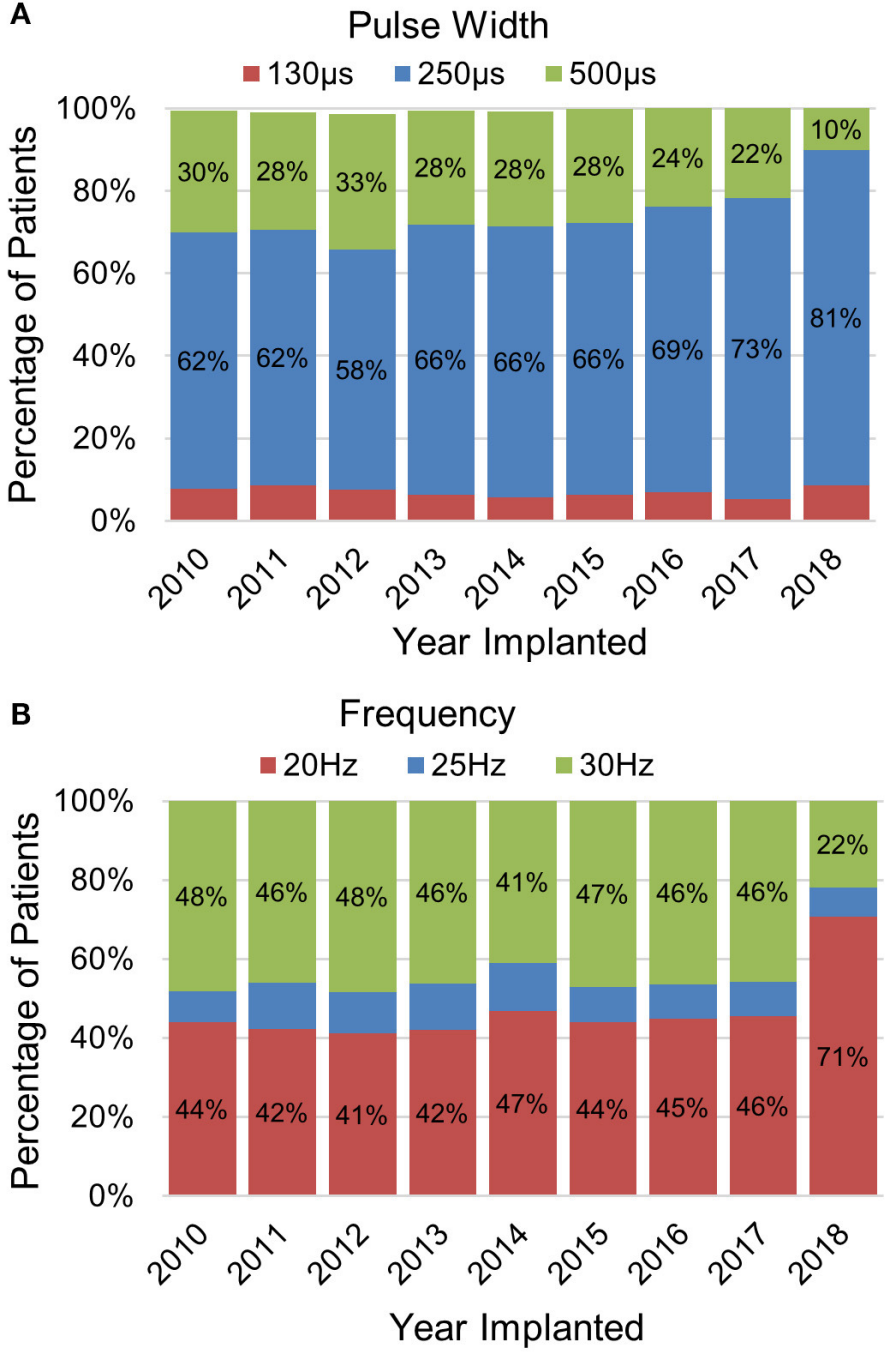
Trends in programming VNS **(A)** frequency and **(B)** pulse width settings. The data represent programming data collected at least 12 months after being implanted with VNS (data on file at LivaNova).

Once activated, VNS delivers a train of pulses with the pulse amplitude being the output current. Other programmed parameters include the pulse width (μs) and signal frequency (Hz) which represents the number of pulses per second. The level of vagus nerve activation is dependent on the combination of these three parameters which exhibit a conventional strength-duration relationship (29). Therefore, shorter pulse widths may require higher output currents to achieve a similar response. Default settings for pulse width and signal frequency are 250 μs and 20 Hz for the model 1000 SenTiva® generator while previous generators defaulted to 500 μs and 30 Hz. The use of lower pulse width and frequency settings of 250 μs and 20 Hz have been reported to result in similar efficacy with improved battery life as compared to higher settings of 500 μs and 30 Hz (26, 28). Lower pulse width and frequency settings are also often programmed to manage stimulation associated side effects (28). Experience with programming VNS, combined with these changes in default settings, have led to a shift in programmed pulse width and frequency settings. For patients implanted with VNS in 2018 with 12 or more months follow-up, ~70% were programmed to 20 Hz and ~80% were programmed to 250 μs (Figure 2). Prior to 2018, the percentage of patients programmed to 20 or 30 Hz were split fairly evenly. Since 2010, the usage of a 250 μs has increased from ~60 to 80%.

Vagus nerve stimulation therapy delivers stimulation at set intervals throughout the day and night. The total percentage of time VNS Therapy® is on for an individual patient is called the duty cycle. Patients typically begin with a normal mode stimulation on time of 30 s and off time of 5 min, equating to a 10% duty cycle. While some patients can achieve benefits at a 10% duty cycle, others will experience additional benefit from increasing the duty cycle, typically by shortening the VNS off time. An example would be to maintain the normal mode stimulation on time of 30 s and decrease the off time to 3 min, equating to a 16% duty cycle. Further increasing the duty cycle and reducing the off time to ≤ 1.1 min, has shown to provide additional benefit to those still having seizures at lower duty cycles (30, 31). Shorter off times are associated with a lower number of AutoStims (19), and for off times <1 min, Autostim cannot be enabled.

Magnet mode output current is typically set 0.25 mA higher than normal mode. When VNS is initially activated, the normal mode output current is 0.25 mA and the magnet mode output current is set to 0.5 mA. As the output current is increased by 0.25 mA, the magnet current is also increased by 0.25 mA. The patient can often feel the magnet mode stimulation at this higher setting and may have minimal but tolerable side effects such as voice changes. Tolerability of the higher magnet mode setting can also be an indication the patient has acclimated to the next step up in dosage. For example, during the titration period, the patient can be asked to swipe the magnet multiple times to prepare for the next increase in output current. The magnet mode is typically programmed with a pulse width of 250 or 500 us and the on time is typically 60 s, although some patients may be set to 30 s or less. Magnet mode stimulation trumps all VNS modes and will deliver stimulation whenever the device is activated using the magnet.

AutoStim works in conjunction with normal and magnet modes. AutoStim output current is typically set 0.125 mA higher than that of normal mode, unless normal mode output current is 2 mA or higher, in which case the AutoStim current should equal the normal mode output current. The AutoStim pulse width is typically set to the same as that of normal mode, most commonly 250 μs, and the on time is typically 60 s, although some patients are set to 30 s. The sensitivity of the heart rate measurement is set in the operating room between 1 and 5, with 1 being the least sensitive. This should be verified prior to activation by comparing the patient's heart rate to the heart rate detected by the generator. An additional setting to be programed is the threshold for AutoStim. This typically starts at 40%, although added benefits may be seen in patients be decreasing the threshold to 30 or 20%. Once the heart rate increases by at least the percentage set as threshold, an AutoStim is delivered. This is followed by the normal mode off time, which can be no less than the AutoStim on time duration. Immediately following an AutoStim, there is an enforced off time equal to the AutoStim on time where an AutoStim cannot be triggered in order to avoid over stimulation of the nerve.

## Advanced Features

**Figure 3:**
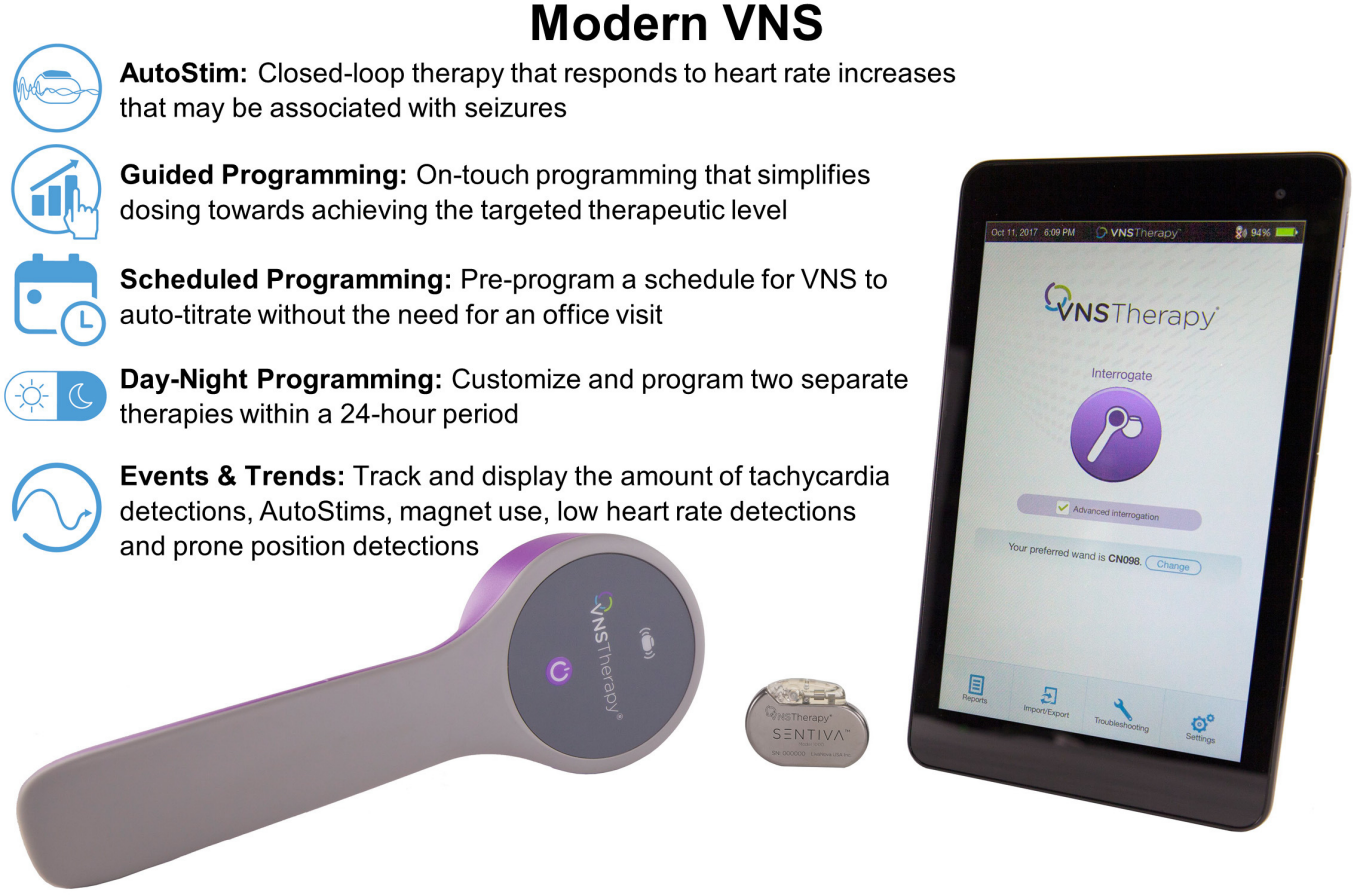
Modern VNS allows additional features to traditional VNS including closed-loop AutoStim, Guided Programming, Scheduled Programming, Day-Night Programming, and Events and Trends data.

The model 1000 SenTiva® generator has advanced features available to simplify dosing, individualize therapy, and collect data (Figure 3). To aid in simplifying and standardizing dosing, Guided Mode is available which allows the programmer to adjust settings with a single button. The steps in output current follow an FDA approved protocol based on published guidelines (28) known as the Standard Protocol (Table 1). A Custom Protocol can be created to adjust pulse width and frequency for each stimulation mode, adjust the normal mode duty cycle, or adjust the output current step size to 0.125 mA. The step size in output current cannot exceed 0.25 mA. Another added benefit of using the Standard or Custom Protocol is the ability to use Scheduled Programming which enables titration without required office visits. With Scheduled Programming, the healthcare provider can schedule the device to auto-titrate up to multiple steps on set days and times. The interval between steps is limited to 0.125 mA every 7 days or 0.25 mA every 2 weeks. This is especially useful for patients who have difficulty making office visits due to distance, limited mobility, or pandemic-related access or travel limitations. If telemedicine visits are an option, these can be set up on the same day or day after the auto-titration to assess tolerability and side effects remotely. It is important for the patient to have the magnet accessible when undergoing scheduled programming in case the patient experiences discomfort with increased levels of stimulation and therapy needs to be turned off.

**Table 1:** Standard protocol dosing steps.

**Step**	**Output current (mA)**		
	**Normal**	**AutoStim**	**Magnet**
1	0.25	0.375	0.50
2	0.5	0.625	0.75
3	0.75	0.875	1.00
4	1.00	1.125	1.25
5	1.25	1.375	1.50
6	1.50	1.62	1.75
7	1.75	1.875	2.00
Frequency:	20 Hz for all modes		
Pulse width:	250 μs for normal and AutoStim		
	500 μs for magnet mode		
Duty cycle:	10% (30-s on, 5-min off)		

The Day/Night Programming feature enables the delivery of different VNS parameters for two different time periods within a 24-h cycle. This feature is not available in Guided Mode and the programmer must be in Manual Mode. This feature is useful to mitigate side effects or provide a higher dose of VNS during certain times of the day or night. For example, if a patient has well-controlled daytime seizures but continues to predominantly have seizures at night, this feature may be used to deliver higher current at nighttime. For patients with obstructive sleep apnea, this feature may be beneficial in scheduling reduced pulse width, frequency and/or current at nighttime. For patients receiving AutoStim who exercise on a regular basis, Day/Night Programming can be used to turn off AutoStim or increase the AutoStim threshold during a specific timeframe to minimize or eliminate AutoStim associated with exercise induced heart rate increases. It is important to note that this setting does not adjust for daylight savings time or changes in time zones.

**Figure 4:**
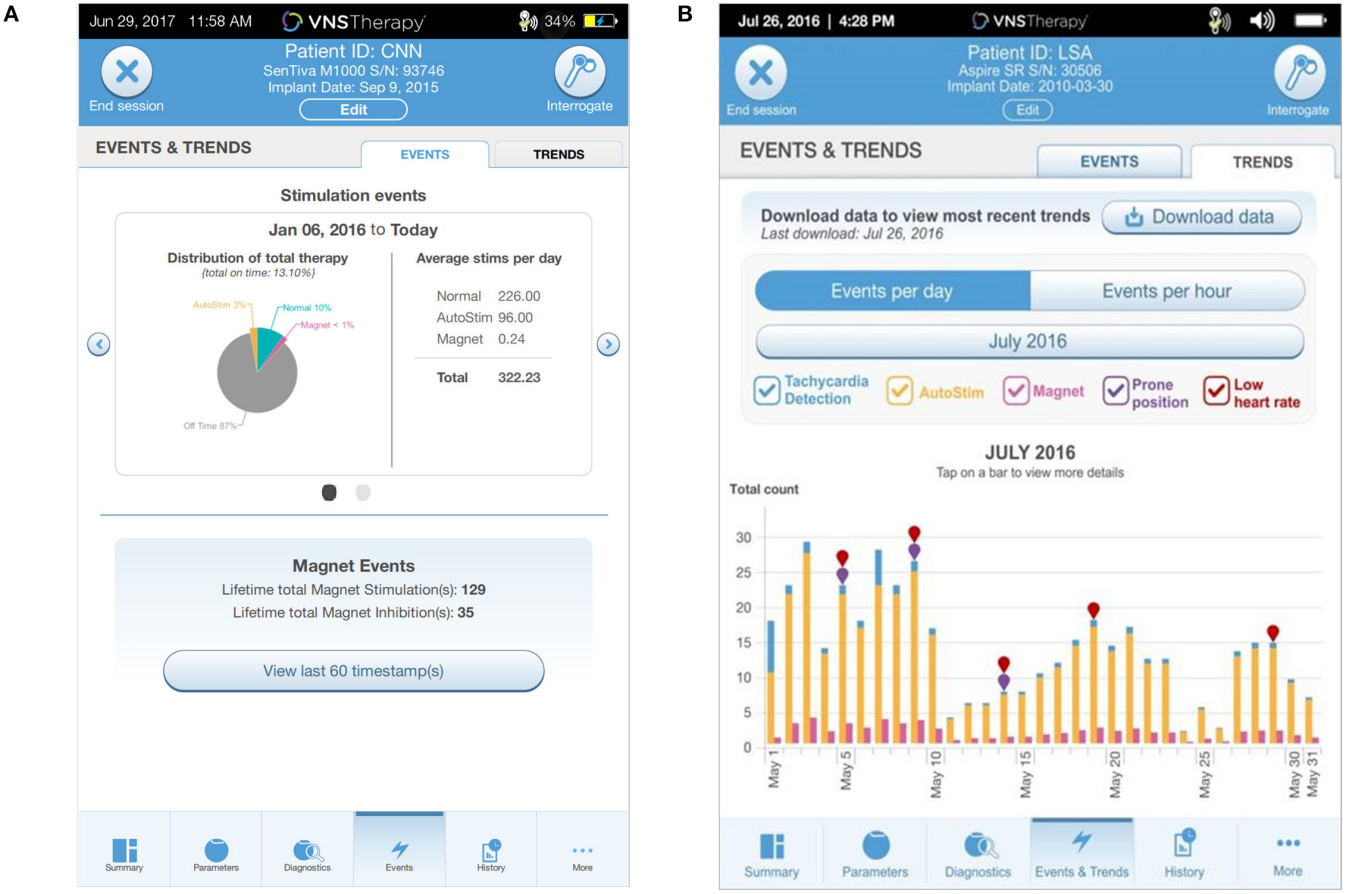
The **(A)** Events tab displays a pie chart showing the distribution of daily Normal, AutoStim, and Magnet stimulations as well as average number of stimulations per day. The **(B)** Trends tab displays daily event counts for tachycardia detections, AutoStims, Magnet mode stimulations, prone position detections, and low heart rate detections.

The programmer has an Events tab to view Events (summary data from recent office visits; Figure 4A) and Trends (daily and hourly trends of data; Figure 4B). Events displays a pie chart showing the daily distribution of normal mode, magnet mode, AutoStim, and Off time, as well as daily average number of stimulations for each mode. Overall duty cycle can also be viewed, combining total amount of time the normal, magnet and AutoStim features are active in a patient. Trends displays the daily or hourly number of tachycardia detections, AutoStims, and magnet mode stimulations. This can help guide future programming and patient education. For example, if the patient or caregiver report magnet activations that are not seen upon interrogation, additional counseling regarding the proper technique to activate the device should be reviewed. If the average AutoStims per day is low, you may want to lower the Autostim threshold to determine if lower thresholds lead to an increase in the average number of AutoStims and an associated decrease in either seizure frequency or duration.

The device can also be set to detect and track low heart rate and prone positioning which can be setup in the tachycardia detection window. The low heart rate threshold can be set to 30, 40, 50, or 60 beats per minute. Turning on prone position detection requires a simple calibration of the accelerometer within the generator. The generator will only sense for a low heart rate detection and prone positioning 7.5 min following an AutoStim or Magnet mode stimulation. A timestamp for these events can then be seen in the events and trends window. Prone position detection can only be used when tachycardia detection is enabled. This feature is currently only used for reporting purposes with no real-time notification or alarm in place when these events are detected. The results can be used to enhance your discussion of Sudden Unexplained Death in Epilepsy (SUDEP) with your patients and caregivers.

**Figure 5:**
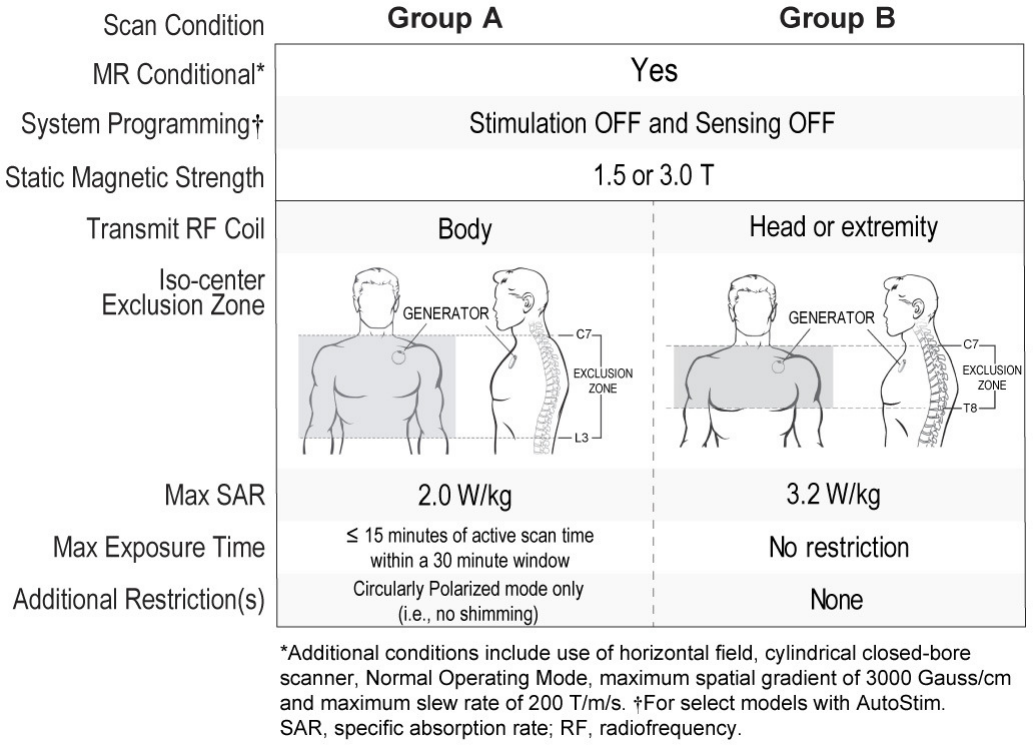
MRI conditions with VNS Therapy^Ⓡ^.

Magnetic resonance imaging (MRI) compatibility has expanded with the latest VNS devices, increasing access to high quality MRI scans. Patients implanted with functional single-pin leads and generator models 103 Demipulse®, 105 AspireHC®, 106 AspireSR®, or 1000 SenTiva® implanted in the typical upper chest location at or above the armpit area (above rib 4) can safely receive scans using a transmit body coil as long as its iso-center is outside C7-L3 (Group A in Figure 5). There are no restrictions on the type of receive coil that can be used. Use of a transmit body coil enables use of high channel count receive-only head coils which can be used to collect high quality MRI or functional MRI brain scans. Older generators, dual-pin generators, broken leads, or atypical generator implant locations are not compatible with transmit body coils and require use of extremity transmit/receive coils with the iso-center outside C7-T8 (Group B in Figure 5).

## Practical Management of VNS Clinics

Development of a dedicated VNS clinic by an APP can lead to improved patient selection, patient education, and development of expertise by a core group of clinicians. Patients who are considered for VNS Therapy® are often presented at a multidisciplinary surgical case conference that includes neurology, neurosurgery, neuroradiology, neuropsychology, psychology, and other experts. Once the team determines the patient is a candidate for VNS, the patient should be scheduled to see the APP in a VNS clinic for a pre-VNS evaluation to discuss risks, benefits, side effects, dosing, and frequency of visits. It is important for the patient and family to understand the importance of follow-up and dosing after implantation because results depend on proper dosing to a therapeutic level (26, 28). The side effect profile of VNS Therapy® is unique in that it is does not cause central nervous system side effects seen with many anti-seizure medications (27). Treatment options that do not cause sedation or cognitive side effects are important to patients. While VNS is an adjunctive treatment along with anti-seizure medications, it is possible at times to reduce polypharmacy when VNS Therapy® is effective. Although few patients are rendered seizure-free with VNS Therapy®, the potential for reduction in seizure frequency, seizure duration, seizure clusters as well as the potential for other improvements in quality of life should be reviewed. Discussion of surgical risks should include risk of infection as well as the rare complication of vocal cord paralysis. Magnetic resonance imaging restrictions should be reviewed and a thorough review of the patient's medical history will help with direct counseling on potential contraindications associated with MRI. Patients with a history of reflux, apnea, arrythmia or bradycardia need to understand the potential for worsening symptoms. Evaluation by a cardiologist is recommended for patients with predisposed dysfunction of cardiac conduction systems.

Patients should be made aware that the generator will need to be replaced periodically as the battery life is typically between 5 and 10 years and depends on the level of VNS settings. More frequent follow-up, such as every 3 months, may be required after the VNS generator has been in place for several years in order to monitor battery life. The lead will be left in place unless there is a serious reason for removal. Removal of the lead and electrode can be done, however there is a risk of damaging the vagus nerve, and generally should not be undertaken unless the benefit of removal clearly outweighs the risk of the procedure. Coordinating a pre-VNS counseling session with an evaluation by neurosurgery allows for a strong handoff to the surgeon with VNS relevant information provided by the APP. It also provides a more comprehensive approach to ensure the patient and guardians have adequate information to make an informed decision about proceeding with VNS placement. Once a patient or guardian elects to move forward with VNS placement, informed consent is obtained, surgery is scheduled, and a follow up visit in the VNS clinic should be scheduled for 2 weeks after implantation. The patient is typically given a magnet kit upon implantation that should be brought to each follow up visit.

At the VNS activation visit, the APP begins by assessing the incisions to ensure they are healing without signs of infection. At the time of activation, patients will often experience a voice change characterized by a deepened tone or a warble to their voice during VNS on time, or the patient may cough at the onset of stimulation. Patients who experience throat clearing or a mild cough often find that the symptoms subside within 1 to 2 days, however this can be a dose limiting side effect in the beginning of therapy. Over time patients tend to better tolerate VNS settings as side effects resolve and further dose titration is possible. Voice changes can also improve with time, but can become more pronounced with subsequent increases in current. Although this is a common side effect, it typically does not limit the ability to titrate therapy. With each dose titration, the APP should monitor for signs of discomfort, coughing, or other intolerable side effects, and be prepared to decrease the dosing parameters if needed. For newly implanted patients, the APP can increase the dose in a stepwise fashion such as that in Table 1 and monitor the patient for side effects during the visit. It is often possible to increase the output current multiple steps during the first visit. If side effects of cough or voice change occur while slowly titrating the dose over a 30 min period, then the APP can step back to the last previously tolerated output current.

Once activated, patients and caregivers should be instructed in the proper use of the magnet. Discussion around the times to use the magnet, frequency of magnet activation for a single event, and the proper technique to turn off the device with the magnet should be reviewed. Those present at the visit should also demonstrate the proper technique to use the magnet. It can be helpful to review magnet activations recorded by the device to see if the patient and family are correctly using the magnet at home. It is also helpful to provide a letter for all caregivers to explain the use of the magnet, as well as magnet restrictions.

Monitoring battery life throughout follow-up is important in order to properly plan for a generator replacement procedure prior to battery depletion. As the battery becomes low, device diagnostics will show the following battery life indicators: Intensified Follow-up Indicator (IFI), Near End of Service (NEOS) and End of Service (EOS) when the battery life is 8–18%, 0–8%, and 0%, respectively. When the battery life indicator is displaying IFI, it is recommended to schedule more frequent follow-up visits with the patient, such as every 3 months, in order to closely monitor battery life. The full benefits from VNS may take years to fully appreciate (7), and benefits may be lost acutely or gradually, and possibly permanently after EOS (32). It is therefore important to weigh all potential benefits of VNS in addition to seizure control when considering generator replacement, including effects on alertness, post-ictal state, cluster seizures, mood, and memory. Generator replacement should be done prior to EOS to ensure long-term treatment. Patients should also be informed that if they experience a sudden change in seizure frequency, decreased perception of stimulation, or loss of other VNS-induced effects after being implanted for several years, the device should be checked to see if the battery is near end of service. For patients with older generation dual-pin leads, a dual-pin compatible generator is required for replacement, such as the model 104 generator. If a dual-pin compatible generator is not available in a specific region, then a lead replacement would be required during the generator replacement procedure. If discontinuation of therapy is being considered due to lack of efficacy or intolerable side effects, VNS Therapy® can be turned off for an extended period of time, such as 6 months, to determine if seizure activity or other potential VNS-induced benefits change. If it is determined that VNS was not beneficial to the patient, the device may remain implanted but with all output current settings programmed to zero, or for patients who prefer, the VNS system can be fully or partially explanted (33, 34).

## Troubleshooting

The benefit of a trained APP is the knowledge to troubleshoot and ability to see patients for urgent visits should problems arise. Difficulties with interrogation can occur. If this arises the battery power of the wand should be assessed and batteries should be replaced if needed. If the battery light on the wand is green, reposition the wand to attempt interrogation. If the Bluetooth connection between the wand and tablet are not adequate, connect the wand to the tablet via the cord provided with the device. In some cases, repositioning of the patient's arm or placing the patient supine is needed to make the device more accessible to the wand.

When interrogating the device, a high or low lead impendence may be detected. This can occur with lead discontinuity, disconnection or fibrosis. If a high impedance error message comes up soon after surgery this could be due to the setscrew or lead pin not being fully inserted into the generator. If a high lead impedance message occurs in an established patient, this could indicate a lead break and all output currents should be turned off. Obtaining an x-ray to visualize the lead is often indicated if there is a high or low lead impedance, however it is possible for the break in the lead to be small and not visible on x-ray.

Other than technical difficulties, an appropriate understanding of managing side effects is imperative for any provider running a VNS clinic. Many dose related side effects, such as reflux, cough, voice change and sleep apnea can be addressed by adjusting the settings. This includes decreasing the output current, signal frequency, pulse width or duty cycle. For patients who are unable to tolerate a higher output current, the duty cycle can be increased and may provide additional benefit. Some patients are bothered by the noticeable difference in normal mode and AutoStim output current. In these cases, AutoStim can be set at the same output current as normal mode. Taping the magnet in place to temporarily turn off VNS Therapy® can be done during eating for patients who note difficulty in swallowing or can be done during singing or public speaking in patients who experience stimulation induced voice alterations. In these situations, the magnet should be removed immediately after eating, singing, or public speaking is complete. It is important to know that no stimulation will occur if the magnet is taped or continually held in place over the VNS. This technique of placing the magnet over the generator can also be used if there is concern the VNS is not tolerated as evidenced by painful stimulation, intense neck pain, or trouble breathing. The magnet can be taped in place until the patient is able to obtain medical attention.

In rare cases, patients have reported discomfort along the track of the lead, but interrogation failed to reveal any abnormality. Intraoperative examination has revealed degradation of the casing around the wire, which caused the patient pain without triggering an abnormal diagnostic. If a patient consistently reports a problem, the provider must consider that there may be something wrong that is not detectable by standard interrogation, and refer the patient back to the surgeon.

Vagus nerve stimulation is an efficacious intervention for DRE, but it will not benefit every patient. There may be times when patients do not feel they have benefitted from the device, and they wish to have it removed. Every center will establish its own criteria for explanting a VNS, but it is reasonable to wait until the patient has had it on reasonably optimized settings for 2 years, and every effort should be made during that time to ensure the best settings the patient can tolerate are achieved. If the patient and care team think VNS removal is warranted due to lack of efficacy, they may wish to consider simply turning off the device for 6 months before explanting it. This gives the patient a chance to see if perhaps the VNS was providing more benefit that previously realized. Tracking cognitive abilities, mood, and quality of life scores during this time would be helpful in determining if the patient notices any deterioration in these metrics without the VNS on.

## Conclusion

Vagus nerve stimulation is a well-established adjunctive treatment in reducing seizure frequency in patients with DRE, and VNS has evolved into a smarter technology over the past decade with the addition of closed-loop stimulation and features the enable guided and scheduled dosing and more individualized therapy. Advanced Practice Providers play a critical role in dedicated VNS clinics which provide value to patients and caregivers through improved access, education, and continuity of care. Continued education on the practical use of traditional and modern VNS features as described in this review is important to maximize benefit to VNS patients.

## Author Contributions

All authors contributed to the first five sections describing VNS Therapy®. The sections PRACTICAL MANAGEMENT OF VNS CLINICS and TROUBLESHOOTING were written exclusively by the advanced practice provider authors BF, JD, EK, and SM.

## Conflict of Interest

SW is an employee of LivaNova PLC. The remaining authors declare that the research was conducted in the absence of any commercial or financial relationships that could be construed as a potential conflict of interest.
